# Evaluation of Antiradical and Anti-Inflammatory Activities of Ethyl Acetate and Butanolic Subfractions of *Agelanthus dodoneifolius* (DC.) Polhill & Wiens (Loranthaceae) Using Equine Myeloperoxidase and Both PMA-Activated Neutrophils and HL-60 Cells

**DOI:** 10.1155/2015/707524

**Published:** 2015-03-02

**Authors:** Rainatou Boly, Thierry Franck, Stephan Kohnen, Marius Lompo, Innocent Pierre Guissou, Jacques Dubois, Didier Serteyn, Ange Mouithys-Mickalad

**Affiliations:** ^1^Institute for Research in Health Sciences (IRSS/CNRST), 03 BP 7192 Ouagadougou 03, Burkina Faso; ^2^Institute of Chemistry B6a, Centre of Oxygen Research and Development (CORD), University of Liege, Sart Tilman, 4000 Liege, Belgium; ^3^Department of Clinical Sciences, Faculty of Veterinary Medicine, University of Liege, B41, Sart Tilman, 4000 Liege, Belgium; ^4^Celabor SCRL, Avenue du Parc 38, 4650 Herve, Belgium; ^5^Laboratory of Pharmacology and Toxicology, University of Ouagadougou, UFR/SDS, 03 BP 7021 Ouagadougou 03, Burkina Faso; ^6^Institute of Pharmacy, Laboratory of Toxicology and Applied Physical Chemistry, Free University of Brussels, Campus de la Plaine, CP 205/01, Boulevard du Triomphe, 1050 Brussels, Belgium

## Abstract

The ethyl acetate and *n*-butanolic subfractions of *Agelanthus dodoneifolius* were investigated for their antioxidant and antimyeloperoxidase (MPO) activities. The reactive oxygen species (ROS) generation was assessed by lucigenin-enhanced chemiluminescence (CL) and dichlorofluorescein- (DCF-) induced fluorescence techniques from phorbol myristate acetate- (PMA-) stimulated equine neutrophils and human myeloid cell line HL-60, respectively. In parallel, the effects of the tested subfractions were evaluated on the total MPO release by stimulated neutrophils and on the specific MPO activity by means of immunological assays. The results showed the potent activity of the butanolic subfraction, at least in respect of the chemiluminescence test (IC_50_ = 0.3 ± 0.1 *µ*g/mL) and the ELISA and SIEFED assays (IC_50_ = 2.8 ± 1.2 *µ*g/mL and 1.3 ± 1.0 *µ*g/mL), respectively. However, the ethyl acetate subfraction was found to be the most potent in the DCF assay as at the highest concentration, DCF fluorescence intensity decreases of about 50%. Moreover, we demonstrated that the ethyl acetate subfraction was rich in catechin (16.51%) while it was not easy to identify the main compounds in the butanolic subfraction using the UPLC-MS/MS technique. Nevertheless, taken together, our results provide evidence that *Agelanthus dodoneifolius* subfractions may represent potential sources of natural antioxidants and of antimyeloperoxidase compounds.

## 1. Introduction

Free radicals, especially reactive oxygen species (ROS), which are produced during the oxidative burst of polymorphonuclear neutrophils (PMNs), are generated by two key enzymes, namely, NADPH oxidase and myeloperoxidase (MPO) [1]. PMNs are thought to produce both extracellular and intracellular reactive oxygen species that may have an important role in the host defense against invading microorganisms [2]. Although ROS have been implicated in the host defense, under adverse circumstances, they may be harmful for host cells or tissues when produced in excess and can oxidize essential biomolecules such as DNA, proteins, and lipids leading to the development of several diseases such as cancer, cardiovascular disease, rheumatism, and neurodegenerative disorders [3].

During the neutrophil degranulation, myeloperoxidase (MPO), a heme-containing peroxidase, is released outside cellular compartment and is tightly linked to both inflammation and oxidative stress. MPO represents the most abundant enzyme stored in the azurophilic granules of neutrophilic granulocytes [4, 5]. MPO plays an important role in the killing of microorganisms that occurs in the phagosome of neutrophils through the formation of hypochlorous acid (HOCl) from the chlorination cycle of the enzyme evolving hydrogen peroxide (H_2_O_2_) and a chloride ion (Cl^−^) [6]. Nevertheless, in conditions of excessive and uncontrolled inflammation, the degranulation as well as release of MPO in the extracellular space has been linked to the pathogenesis of various diseases such as atherosclerosis, multiple sclerosis, and cancer [5]. Therefore, compounds with radical scavenging and antimyeloperoxidase activities may be relevant in the treatment of diseases where free radicals and MPO are implicated.

Natural antioxidants are increasingly preferred since they have better antioxidant activity and their natural origin makes them more attractive comparatively to synthetic antioxidants [7]. Despite the fact that the nonsteroidal anti-inflammatory drugs (NSAIDs) have beneficial effects in the treatment of inflammatory related diseases, their several adverse effects such as gastric/duodenal ulceration and renal failure are a serious inconvenient in their clinical use [8].

Considering the large use of medicinal plants throughout the world [9] for the treatment of various ailments, the need to prove their real activity or to provide ethnomedical information evidence is required. Plants produce several secondary metabolites with important functions and phytochemicals such as phenolics, alkaloids, and terpenoids were reported to be responsible of the anti-inflammatory activity [10]. The anti-inflammatory activity of phytochemicals involves different mechanisms of action such as antioxidant and radical scavenging properties, modulation of the cellular activities of inflammatory cells, and proinflammatory enzyme activities [10]. Among plants phytoconstituents, phenolics were the most abundant and those also reported to have scavenging properties based on various methods [11, 12]. Our previous works have shown that crude fractions from* Agelanthus dodoneifolius* plant displayed interesting antioxidant activities that were related to their polyphenolic contents [13]. As those polyphenol components are very important at inducing antioxidant and anti-inflammatory effects, we were particularly interested to investigate which active component belonging to the subfraction is responsible of the antioxidant/antiradical activity.

The use of plants as therapeutic agents may have several goals such as the isolation or the production of bioactive compounds with new structures [9]. However, besides being time-consuming with high solvent and low recoveries, sometimes, the process of isolating could produce a compound less active comparatively to the whole plant or some bioactive compounds might inadvertently be excluded [14]. Thus, the use of crude and/or standardized extracts is about to crystallize further efforts from the scientific community [15]. Our work is in this context of standardization through purification of* Agelanthus dodoneifolius* (Loranthaceae) extracts, a plant used in Burkina Faso folk medicine for the treatment of cardiovascular and respiratory diseases, stomachache, and wounds. The present study aims to evaluate the antiradical and antimyeloperoxidase activities of the ethyl acetate and butanolic subfractions obtained from the mother extracts after fractionation on a column chromatography.

## 2. Materials and Methods

### 2.1. Chemicals and Reagents

Analytical-grade diethyl ether,* n*-butanol (Merck, VWRI, Leuven, Belgium), methanol, and ethyl acetate (Chem-Lab, Zedelgem, Belgium) were used in the extraction procedure. Sodium chloride (NaCl), potassium chloride (KCl), calcium chloride (CaCl_2_), ammonium acetate, potassium hydroxide, sodium hydroxide, acetic acid, ethanol, hydrogen peroxide (H_2_O_2_), dimethyl sulfoxide (DMSO), ethylene diamine tetra-acetic acid, disodium salt (EDTA-Na_2_H_2_), trypan blue, and tween-20 were supplied by Merck (VWRI, Leuven, Belgium). Paranitrophenyl phosphate, bis(N-methylacridinium) nitrate (lucigenin), phorbol 12-myristate 13-acetate (PMA), Percoll, sodium carbonate, and gallic acid were all purchased from Sigma (St. Louis, USA). Catechin and quercetin were from ChromaDex (LGC Standard, France). Amplex Red was purchased from Molecular Probes (Invitrogen, Merelbeke, Belgium). Bovine serum albumine (BSA) and horseradish peroxidase (HRP) were supplied by Roche (Germany). 2′,7′-Dichlorofluorescin-diacetate (DCFH-DA) was obtained from Eastman Kodak (Rochester, NY, USA). All the solutions were prepared with deoxygenated MilliQ water or ultrapure water (Easy Pure UV purification system, Barnested/Thermolyne, Dubuque, USA).

### 2.2. Plant Material

*Agelanthus dodoneifolius* leaves were collected in the region of Ouagadougou between October and November 2005 from* Vitellaria paradoxa* CF Gaertn (Sapotaceae). The plant materials were authenticated and a voucher specimen (numbers 01 and 02) was lodged in the herbarium of the Plant Ecology and Biology Laboratory, University of Ouagadougou.

### 2.3. Extraction Procedure

The shade dried and powdered leaves of* Agelanthus dodoneifolius* (200 g) were boiled in methanol for 15 min. The methanolic extract was evaporated under reduced pressure using a rotavapor. The residue was resuspended in boiling water and successively exhausted with solvent of increasing polarity: diethyl ether (4 × 150 mL), ethyl acetate (5 × 150 mL), and* n*-butanol (4 × 150 mL). The extraction yielded (i) 375.4 mg of diethyl ether extract, (ii) then 1.2 g of ethyl acetate extract, and, finally, (iii) 3.1 g of butanolic extract.

Ethyl acetate extract (1.07 g) was subjected to silica-gel column chromatography (55 g, 60 Å) using solvent systems: EFW (ethyl acetate/formic acid/water: 90/5/5). The fractions were collected and combined after monitoring by TLC. Six fractions were obtained: fraction I: 513 mg; fraction II: 330 mg; fraction III: 124.3 mg; fraction IV: 34.1 mg; fraction V: 58.6 mg; fraction VI: 10 mg. Fraction I was applied to silica-gel column chromatography with the solvent systems EFW: 60/5/5. After elution and monitoring by TLC, 4 fractions (FIa–FId) were obtained. Ethyl acetate subfraction 1 (E1, 300 mg) was obtained from FIa fraction after precipitation in the solvent systems: ethyl acetate/chloroform (2/1).

**Figure 1:**
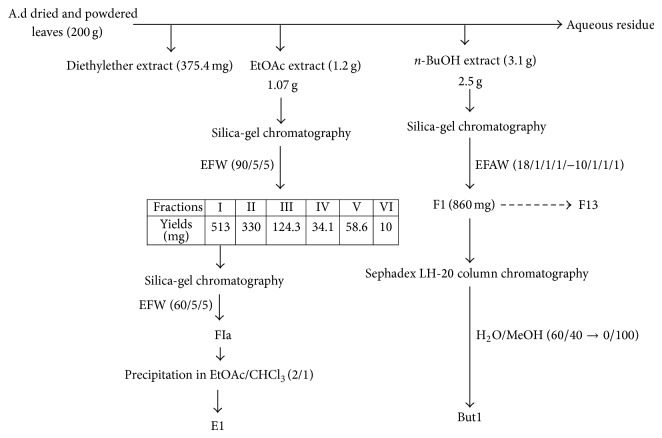
Scheme of the ethyl acetate and butanolic extracts fractionation. A.d:* Agelanthus dodoneifolius*; EFW: ethyl acetate-formic acid-water; EFAW: ethyl acetate-formic acid-acetic acid-water; E1: ethylacetate subfraction 1; But1: butanolic subfraction 1.

Butanolic fraction (2.5 g) was chromatographed on a silica-gel column chromatography (75 g, 60 Å) using gradient solvent systems: EFAW (ethyl acetate/formic acid/acetic acid/water: from 18/1/1/1 to 10/1/1/1). 13 fractions (F1–F13) were collected and combined after monitoring by TLC. Fraction F1 (860 mg) afforded butanolic subfraction 1 (But1, 170 mg) after elution with water/methanol mixture whose proportion varies from 60/40 to 0/100 on a Sephadex LH-20 column chromatography (27 g, 25–100 *μ*m) (Figure 1).

### 2.4. Isolation of Equine Neutrophils

Blood samples were drawn from healthy horses by venous puncture into vacutainer tubes with EDTA (1.6 mg/mL blood) as anticoagulant. Horses were fed and bred in identical conditions and were not subjected to any medical treatment (Faculty of Veterinary Medicine, University of Liege, Belgium). The neutrophils were isolated at room temperature (18–22°C) by centrifugation on a discontinuous Percoll density gradient according to the method of Pycock et al., [16], as detailed elsewhere [17]. The cell suspension contains more than 90% of neutrophils and displays a viability > 90% as established by the trypan blue exclusion test. Each batch of neutrophils was prepared from 90 mL of blood from one horse. The cells were used within 4 h after isolation and each assay was performed in triplicate. Each experiment was repeated at least three times with different cell batches collected from different horses.

### 2.5. Cell Culture

The human myeloid cell line HL-60, obtained from the American Type Culture Collection (ACCT, USA), was grown in Iscove's Modified Dulbecco's Medium (IMDM) supplemented with 20% (v/v) fetal calf serum, 100 U/mL penicillin/streptomycin, 1.25 mg/mL amphotericin B, and 2 g/L NaHCO_3_ in 50 mL flasks at 37°C in a 5% CO_2_ humidified atmosphere. The cells were cultured and fed two to three times per week to maintain a log phase growth and twice a week, they were centrifuged and resuspended in fresh IMDM. Before each experiment, cells were counted with Bürker cell counting chamber (Briare, France) to reach the cell density of 10^6^ cells/mL with a viability of > 95% as assessed by the exclusion of trypan blue dye.

### 2.6. Preparation of Neutrophil Stimulating Agent

Phorbol 12-myristate 13-acetate (PMA) was dissolved (1 mg/mL) in DMSO and aliquots were kept at −20°C. Just prior to use, 990 *μ*L of ultrapure water was added to the aliquot to obtain a stock solution of 16 *μ*M PMA with 1% DMSO. In the assays, PMA was used at the final concentration of 0.8 *μ*M and 0.05% DMSO. For the stimulated neutrophils, parallel assays were performed with cell suspensions in PBS alone or PBS in 0.05% DMSO.

### 2.7. Preparation of the Samples and Standard

The ethyl acetate and butanolic subfractions were solubilized in DMSO (final concentration in each experiment did not exceed 1%) and were used at final concentrations of 50, 25, 10, 5, 2.5, 1, 0.5, and 0.1 *μ*g/mL for the lucigenin chemiluminescence assay (CL), the release of total MPO by stimulated equine neutrophils, and the measurement of active MPO by SIEFED. Gallic acid was solubilized in DMSO at final concentrations of 12.8, 8.5, 4.3, 1.3, 0.9, and 0.4 *μ*g/mL in these three tests.

For the ROS detection with DCFH-DA with HL-60 cells, samples and the standard gallic acid were used at final concentrations of 10, 5, and 1 *μ*g/mL.

### 2.8. Evaluation of ROS Production by Stimulated Equine Neutrophils

ROS production by stimulated neutrophils was assessed by lucigenin-dependent chemiluminescence (CL), according to a method adapted from Benbarek et al. [18] and described elsewhere [13, 17]. Neutrophil suspensions (1 × 10^6^ cells) were stimulated with PMA in the presence of increasing final concentrations of the extract in a 96-well microtiter plate (White Combiplate 8, Thermo Labsystems) and the CL response was monitored for 30 min at 37°C (Fluoroscan Ascent FL, Thermo Scientific, Belgium) and expressed as the integral value of the total CL emission. A control assay set as 100% of the CL was performed with stimulated neutrophils incubated with DMSO. An additional control was also performed with nonstimulated neutrophils. The final volume including cell suspension, reagents, and extract reached 200 *μ*L.

### 2.9. Evaluation of ROS Production by Stimulated HL-60 Monocytes

The method, based on a fluorometric assay, was adapted from Amado et al., [19]. Briefly, HL-60 cells (1 × 10^6^ cells/mL) were incubated (45 min, 37°C in the dark) in 24-well microtiter plates with 20 *μ*L of a 1 mg/mL DCFH-DA stock in ethanol. Then, the content of each well was transferred into a 5 mL tube for centrifugation (335 ×g, 10 min, 37°C). After removing the supernatant, cells were subsequently resuspended in HBSS buffer and transferred to the wells. After treatment with different concentrations (1, 5, and 10 *μ*g/mL) of the subfractions and gallic acid, 10 *μ*L of HRP (30 *μ*g/mL) and 30 *μ*L of PMA (0.8 *μ*M) were added to the cell suspension. The generation of the fluorescent product DCF was followed in an automated plate fluorescence reader (Fluoroskan Ascent FL, Fischer Scientific, Belgium) for 30 min at 37°C using an excitation wavelength of 485 nm and emission wavelength of 555 nm. The ability of the tested subfractions to inhibit ROS production by activated HL-60 cells was compared to a control assay set as 100% of the ROS induced fluorescence. Control was performed with PMA activated cells in the presence of DMSO. Another control (NA, not activated) was made with cells in the absence of both PMA and test materials to measure the basic ROS production.

### 2.10. Neutrophil Degranulation Assay

MPO released by equine neutrophils stimulated with PMA was assessed by an original ELISA assay raised against equine MPO as previously reported [13, 20] and distributed by BioPtis (Liège, Belgium). Briefly 1 × 10^6^ cells in 1 mL PBS buffer were stimulated with PMA in the presence of the extract for 30 min at 37°C and further centrifuged 10 min at 600 ×g. The supernatants were collected and diluted 200 times in the dilution buffer of the kit for total MPO assay. The absorbance read at 405 nm with the Multiscan Ascent (Thermo Scientific, Belgium) is proportional to the content of MPO released by neutrophils. The control assay set as 100% of MPO released was performed with PMA-stimulated neutrophils in the presence of DMSO. An additional control was performed with the supernatant of nonstimulated neutrophils (10 *μ*L of PMA was replaced by 10 *μ*L of a solution consisting of 10 *μ*L de DMSO and 990 *μ*L of MilliQ water).

### 2.11. Measurement of Active MPO by SIEFED

MPO activity was measured by SIEFED developed for the specific detection of active equine neutrophil MPO [21]. The method proceeds in three steps. Firstly, MPO was extracted from a solution or a biological sample by specific immobilized antibodies (immunoextraction step). Secondly, a series of washings eliminates unspecifically bound compounds or interfering substances. Thirdly, MPO enzymatic activity was revealed by using H_2_O_2_ (10 *μ*M) as substrate, Amplex Red (40 *μ*M) as fluorogenic electron donor, and nitrite (10 mM) as enhancer of the reaction. The activity of MPO transforms Amplex red into a highly fluorescent compound, resorufin, and after incubation (30 min at 37°C in the dark), the fluorescence emission was read (Fluoroscan Ascent FL, Thermo scientific,*λ* excitation, 544 nm;*λ* emission, 590 nm). The control assay set as 100% MPO activity was performed with purified MPO in the presence of DMSO.

### 2.12. UPLC-MS/MS Analysis

The UPLC fingerprint profile was established for the ethyl acetate subfraction and performed on an Acquity UPLC system (Waters, Milford, MA, USA) connected to a photodiode array detector (PDA) and a tandem quadrupole (TQD) mass spectrometer (Waters). Detection was set at 280 nm. The TQD mass spectrometer was equipped with a Z-spray electrospray ionization (ESI) interface; analyses were performed as a single run in a negative mode. MassLynx software (version 4.0, Waters) was used for instrument control and for data acquisition and processing. The subfraction was dissolved in methanol and injected onto an Acquity UPLC BEH Shield RP18 column (2.1 × 100 mm, 1.7 *μ*m; Waters). A gradient elution adapted from an HPLC method [22] was developed for analysis. Gallic acid and catechin were used to act as marker components in the standardization of the ethyl acetate subfraction. Both these compounds were quantified in the subfraction using an external standard method. The identification of polyphenols was carried out using their retention times combined with comparisons of the spectroscopic and spectrophotometric data to those of the standards.

### 2.13. Statistical Analysis

Data were processed and analyzed using the Graph Pad Prism software for Window version 5.0 (GraphPad software, San Diego, CA, USA). Experiments were done in triplicate except for the H_2_DCF-DA fluorescence assay where experiments were done in duplicate. All data are presented as mean ± standard deviation (SD). The IC_50_ values were calculated by a nonlinear regression analysis and an unpaired *t*-test with Welch's correction was used to compare the IC_50_ values of the subfractions. Differences among groups were analyzed by a one-way ANOVA followed by Dunnett's posttest. Differences were significant with a *P* value < 0.05.

## 3. Results and Discussion

### 3.1. Effect of the Ethyl Acetate and Butanolic Subfractions on the ROS Production

Recently we have shown that butanolic and ethyl acetate fractions of* Agelanthus dodoneifolius* exhibited excellent anti-inflammatory and antioxidant properties that were attributed to their polyphenol components [13]. However, these interesting activities were not properly correlated to a specific type of polyphenols contained in each tested fraction. To better understand which polyphenol compound determines the activity, we have designed a new study allowing us to isolate and identify subfractions. The idea behind this work was to know exactly the nature of the component that is responsible for the activity of each tested subfraction.

**Figure 2:**
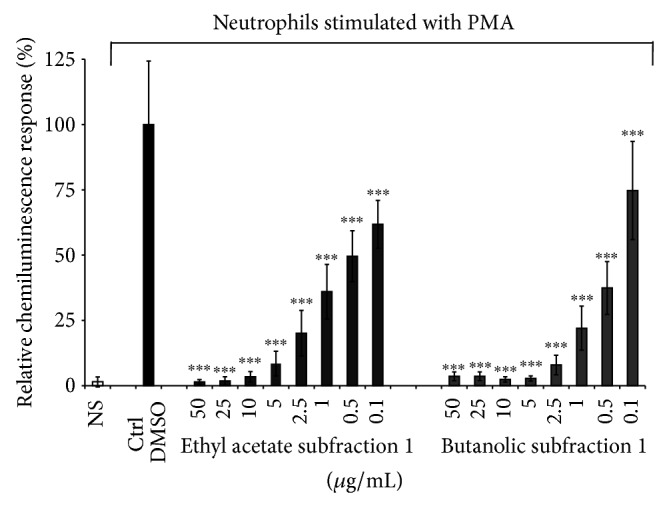
Lucigenin-enhanced chemiluminescence assay. Effect of* Agelanthus dodoneifolius* subfractions on the chemiluminescence response of ROS generated by PMA-treated neutrophils cells. Data are expressed as mean ± SD of three parallel experiments. ^***^ *P* < 0.0001 indicates significance compared to control solutions (one-way ANOVA followed by Dunnett's posttest). NS: not stimulated cells. The IC_50_ values were 0.4 ± 0.2^a^ and 0.3 ± 0.1^a^ *μ*g/mL, respectively, for the ethyl acetate and butanolic subfraction. The IC_50_ value of gallic acid was 0.4 ± 0.1^a^ *μ*g/mL. The letter (a) means that the IC_50_ values were statistically similar (unpaired *t*-test with Welch's correction).

The antioxidant properties of plants, particularly their radical scavenging activities, have been intensively investigated in recent years [23]. The antiradical activity of compounds is related to their susceptibility to react with free radical whereas antioxidant activity refers to an ability to inhibit oxidation processes [24]. There are various methods to determine the free radical scavenging effect. In this study, we assessed the antioxidant and/or free radical scavenging activity of* Agelanthus dodoneifolius* subfractions by using both lucigenin-enhanced chemiluminescence assay and fluorescence-based method using 2′,7′-dichlorofluorescin-diacetate (DCFH-DA) as fluorescent probe. Isolation of antioxidant compounds from plant materials depends mainly on the nature of extracting solvent. Solvent such as water, acetone, ethylacetate, methanol, and* n*-butanol and their mixtures are frequently used for polyphenols extraction from a plant matrix [25] and as described in Figure 1, we used some of these solvents for extraction (liquid-liquid extraction), separation, and purification of* Agelanthus dodoneifolius* fractions. Both ethyl acetate and butanolic subfractions significantly inhibited the oxidation of lucigenin in a concentration dependent manner (Figure 2).

**Figure 3:**
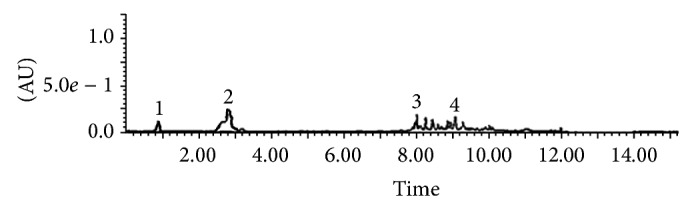
UPLC-UV chromatogram profile of the ethyl acetate subfraction acquired at 280 nm with the photodiode array detector. The subfraction was solubilized in methanol and injected onto an Acquity UPLC BEH Shield RP18 column (2.1 × 100 mm, 1.7 *μ*m; Waters). Peaks 1: gallic acid; 2: catechin; 3: unknown; 4: unknown.

The lowest IC_50_ value was found for the butanolic subfraction (IC_50_ = 0.3 ± 0.1 *μ*g/mL) but was statistically similar to that obtained with the ethyl acetate subfraction (IC_50_ = 0.4 ± 0.2 *μ*g/mL) and gallic acid (IC_50_ = 0.4 ± 0.1 *μ*g/mL). Lucigenin is a well-known luminescent probe that is widely used for its high specificity towards superoxide radical [26]. Our results strongly suggest that* Agelanthus dodoneifolius* subfractions are potential scavengers of superoxide radical, arising from the NADPH oxidase activation, and so that constitutes the first step in the generation of most of the other ROS [27]. Polyphenols such as flavonoids are known to inhibit enzymes responsible for superoxide anion production such as xanthine oxidase, protein kinase C, and NADPH oxidase [28]. It has been suggested that the health benefits afforded by the consumption of fruits, legumes, vegetables, and whole grains are mainly due to the presence of natural antioxidants such as ascorbic acid, carotenoids, tocopherols, and phenolic compounds which represent the most important class [25, 28, 29]. The significant effect of the ethyl acetate and* n*-butanol subfractions is related to their content of polyphenols compounds as demonstrated by the UPLC-MS/MS analysis (Figure 3).

Gallic acid and catechin which were identified through comparisons of their retention times and their spectroscopic and spectrophotometric data compared to those of the standards were found to be present in the ethylacetate subfraction. The amounts of gallic acid and catechin in the ethyl acetate subfraction were 1.63% and 16.51%, respectively, while they were lower (0.09% gallic acid) and undetectable for catechin in the* n*-butanol subfraction. Both of these phenolic compounds are well known for their antiradical properties [30].

**Figure 4:**
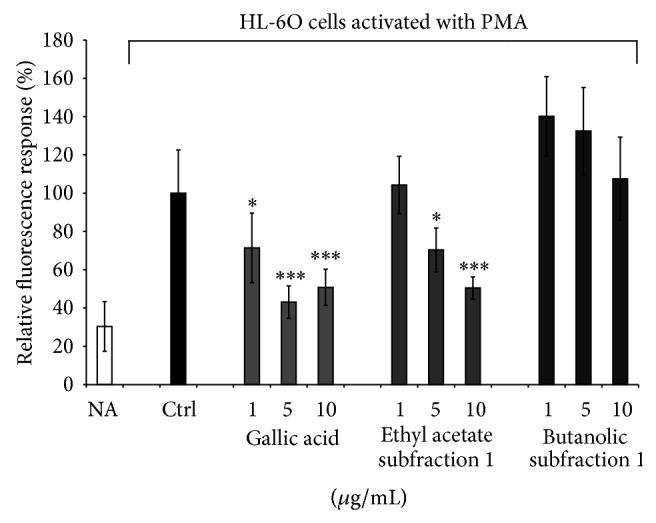
Dichlorofluorescin (DCF) assay. Effect of* Agelanthus dodoneifolius* subfractions on DCF production in PMA-treated HL-60 cells. Data are expressed as mean ± SD of two parallel experiments. ^***^ *P* < 0.0001 or ^*^ *P* < 0.01 indicate significance compared to control solutions (one-way ANOVA followed by Dunnett's posttest). NA: not activated cells.

Interestingly, the ethyl acetate subfraction was more potent at inhibiting the ROS-induced fluorescence from PMA-treated HL-60 cells while the butanolic one failed as shown in Figure 4.

The ethyl acetate subfraction dose dependently decreased the fluorescence intensity of DCF, reaching about 50% of inhibition at the highest concentration. When gallic acid was used instead of the tested subfraction, a dose-dependent inhibition was observed at both 1 and 5 *μ*g/mL final concentrations. Surprisingly, at the highest concentration (10 *μ*g/mL) a less pronounced inhibitory effect was seen for gallic acid. If the two subfractions exhibited a good antioxidant activity with the luminescence assay, by fluorescence it appears that butanolic subfraction rather enhanced the ROS-induced fluorescence, thus behaving as prooxidant agent. When we compare data from both techniques, one can state that the lack of inhibitory effect seen in fluorescence can be explained by a possible presence of other components within the butanolic subfraction which may either fluoresce to the same wavelength that DCF or interfere by another mode of action and amplify the ROS-induced fluorescence. On the other hand, numerous studies report that 2′,7′-dichlorofluorescin-diacetate (H_2_DCF-DA), a peroxide-sensitive fluorescent probe, has been extensively used as a marker for oxidative stress [26, 31]. Contrary to lucigenin whose does not permeate cells, the H_2_DCF-DA can readily diffuse into cells where it is hydrolyzed by intracellular esterases to 2′,7′-dihydrodichlorofluorescin (H_2_DCF), a nonfluorescent derivative. H_2_DCF is oxidized to the highly fluorescent dye 2′, 7′-dichlorofluorescein (DCF) in the presence of ROS [2]. Furthermore, H_2_DCF-DA is more sensitive to H_2_O_2_, ONOO^−^, and ^•^OH and is not suitable for NO, HOCl, or O_2_^•−^ measurement in biological systems [31, 32]. Another explanation of the good inhibitory effect of the ethyl acetate subfraction might be related to their polyphenol contents that have the ability to cross cell membranes and inhibit the intercellular ROS-induced fluorescence while the results obtained with the* n*-butanol subfraction could be explained by the presence of compounds which do not interact with cell membrane and enter into the cells or that fluoresce spontaneously.

### 3.2. Effect of the Ethyl Acetate and Butanolic Subfractions on Neutrophil Degranulation and MPO Activity

**Figure 5:**
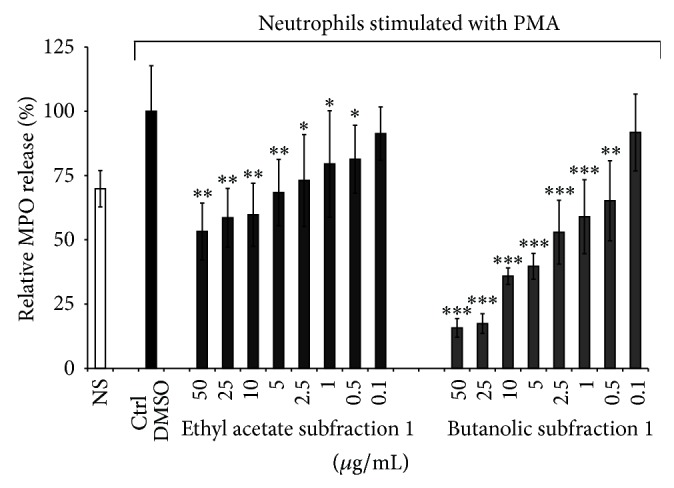
Effect of* Agelanthus dodoneifolius* subfractions on the degranulation of MPO assessed by an ELISA assay. Data are expressed as mean ± SD of three parallel experiments. ^***^ *P* < 0.0001 or ^**^ *P* < 0.001 or ^*^ *P* < 0.01 indicates significance compared to control solutions (one-way ANOVA followed by Dunnett's posttest). NS: not stimulated cells. The IC_50_ values were > 50^a^ and 2.8 ± 1.2^b^ *μ*g/mL, respectively, for the ethyl acetate and butanolic subfraction. The IC_50_ value of gallic acid was > 25^a^ *μ*g/mL. Values not sharing the same letter (a, b) mean that the IC_50_ values were significantly different (unpaired *t*-test with Welch's correction).

Myeloperoxidase (MPO) represents the most abundant proinflammatory enzyme whose release may be associated in the pathogenesis of several diseases. In our study, MPO was used as marker of stimulated neutrophil degranulation. As shown in Figure 5, the* n*-butanolic subfraction has a better inhibitory effect on the release of MPO compared to that of the ethyl acetate subfraction and gallic acid.

When the half maximal inhibitory concentrations were compared, the inhibitory potency decreased in the following order:* n*-butanolic subfraction (IC_50_ = 2.8 ± 1.2 *μ*g/mL) > gallic acid (IC_50_ > 25 *μ*g/mL), data from previous work [13] ≥ ethyl acetate subfraction (IC_50_ > 50 *μ*g/mL). Contrary to our study, Kroes et al. [33] had previously reported an efficient inhibitory effect of gallic acid on MPO release by using another neutrophils activator: zymosan which was different in our model based on PMA stimulation.

We were unable to identify the main compounds of the butanolic subfraction, perhaps because of a lack of standard, but on the basis of chemical tests and preliminary mass spectrometric data (data not published), we hypothesized that butanolic subfraction contains proanthocyanidins, a group of polymerized polyphenols usually referred to as condensed tannins. It was reported that the anti-inflammatory activity of proanthocyanidins might result from the inhibition of the release of proinflammatory factors such as ROS and myeloperoxidase [12, 34]. Moreover, polyphenols such as flavonoids are well known anti-inflammatory compounds and their anti-inflammatory mechanism may involve the inhibition of degranulation [35, 36]. Other studies have reported the inhibitory effect of phenolic compounds on the neutrophil degranulation and it has been shown that flavonoids such as quercetin and rutin were potent inhibitors [10, 37]. In addition, these two compounds were found to be present in* Agelanthus dodoneifolius* leaves as reported previously [13].

Interestingly, the butanolic subfraction had similar activity to that of the mother extract while the ethyl acetate subfraction is less active than the mother ethyl acetate extract [13]. These results suggest that the better activity of the mother ethyl acetate extract is probably due to a combination of its different compounds, and so the loss of activity in the subfraction could be attributed to the purification process.

**Figure 6:**
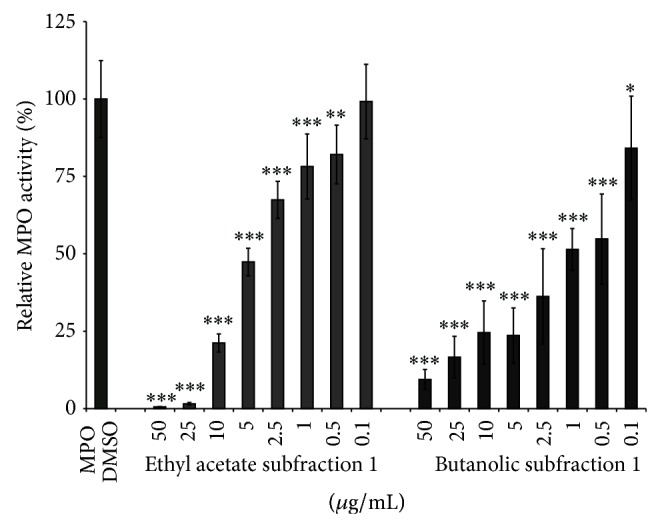
Effect of* Agelanthus dodoneifolius* subfractions on MPO activity measured by SIEFED. MPO was used as marker. Data are expressed as mean ± SD of three parallel experiments. ^***^ *P* < 0.0001 or ^**^ *P* < 0.001 or ^*^ *P* < 0.01 indicates significance compared to control solutions (one-way ANOVA followed by Dunnett's posttest). The IC_50_ values were 3.7 ± 0.5^a^ and 1.3 ± 1.0^b^ *μ*g/mL, respectively, for the ethyl acetate and butanolic subfractions. The IC_50_ value of gallic acid was 1.1 ± 0.2^b^ *μ*g/mL. Values with the same letter (a, b) mean that the IC_50_ values were statistically similar (unpaired *t*-test with Welch's correction).

Thereafter, the ethyl acetate and butanolic subfractions were investigated on MPO activity by SIEFED technique [21] that allows finding compounds which interact directly with the enzyme. The results reported in Figure 6 indicate that* Agelanthus dodoneifolius* subfractions had a dose-dependent inhibitory activity on MPO activity compared to the control sample.

The IC_50_ value for the butanolic subfraction (1.3 ± 1.0 *μ*g/mL) was lower than that of the ethyl acetate subfraction (IC_50_ = 3.7 ± 0.5 *μ*g/mL). It is interesting to note that butanolic subfraction exhibits a better activity than the mother extract while ethyl acetate subfraction and its mother fraction exhibit comparable activities [13]. Since myeloperoxidase plays an important role in the pathogenesis of some inflammatory disorders, a therapeutic goal is to find nontoxic natural compounds that could modulate the activity of this enzyme. The significant activity of* Agelanthus dodoneifolius* subfractions on MPO activity is related to their chemical composition. Polyphenols are the most numerous group of these subfractions and previous studies have demonstrated their ability to inhibit myeloperoxidase activity [38, 39]. Indeed, polyphenols such as quercetin, gallic acid, and proanthocyanidins were reported to inhibit MPO [34, 38, 40].

## 4. Conclusions

Based on our previous study on* Agelanthus dodoneifolius*, a plant used in Burkina Faso folk medicine for the treatment of various ailments, we evaluated the antiradical and anti-inflammatory properties of* Agelanthus dodoneifolius* ethylacetate and butanolic subfractions. The results demonstrate that the ethyl acetate and* n*-butanol subfractions have a significant antiradical and anti-inflammatory potential. The butanolic subfraction exhibited strong potential not only in terms of lucigenin-based chemiluminescence assay but also towards both myeloperoxidase release and activity. On the other hand, the ethyl acetate subfraction exhibited a potent activity in the H_2_DCF-DA fluorescence assay. These significant properties are related to their content in polyphenols compounds as it was shown with the UPLC-MS/MS analysis. Furthermore, the scavenging of free radical and anti-inflammatory properties of polyphenols are well documented in the literature. However, more studies are still needed to isolate and identify the main compounds of the* n*-butanolic subfraction as well as to investigate and confirm on an* in vivo* model the biological activity of* Agelanthus dodoneifolius* subfractions.
